# The Association between Cardio-metabolic and hepatic indices and anthropometric measures with metabolically obesity phenotypes: a cross-sectional study from the Hoveyzeh Cohort Study

**DOI:** 10.1186/s12902-023-01372-9

**Published:** 2023-05-29

**Authors:** Mehrnoosh Zakerkish, Azamsadat Hoseinian, Meysam Alipour, Seyed Peyman Payami

**Affiliations:** 1 Diabetes research center, Health research institute, Ahvaz Jundishapur University of Medical Sciences, Ahvaz, Iran; 2 Department of Nutrition, Shoushtar Faculty of Medical Sciences, Shoushtar, Iran

**Keywords:** Cardiovascular, Hepatic steatosis, Metabolically healthy obesity, Visceral Adiposity Index, Hoveyzeh

## Abstract

**Background:**

This study aimed to compare the cardio-metabolic, anthropometric, and liver function indices among metabolic obesity phenotypes.

**Methods:**

In this cross-sectional study, which was carried out in Hoveyzeh, Khuzestan Province, Iran, a total of 7,464 individuals (male: 2859, female: 4605), were recruited and classified into four groups, based on Body Mass Index (obese, BMI ≥ 30 kg/m^2^; non-obese, BMI = 18.5–29.9 kg/m^2^) and the National Cholesterol Education Program and Adult Treatment Panel (NCEP ATP) III criteria (Healthy group, ≤ 1 of the criteria; Unhealthy group, ≥ 2 of the criteria), as follows: Metabolically Healthy Non-Obese (MHNO, 28.14%), Metabolically Unhealthy Non-Obese (MUNO, 33.06%), Metabolically Healthy Obese (MHO, 6.54%), and Metabolically Unhealthy Obese (MUO, 32.26%). Anthropometric indices (Waist/Hip Ratio (WHR), Waist/Height Ratio (WHtR), Body Adiposity Index (BAI), Visceral Adiposity Index (VAI), and Weight adjusted Waist Index (WWI)), cardio-metabolic indices (Atherogenic Index of Plasma (AIP), Lipid Accumulation Product (LAP), Cardio-Metabolic Index (CMI), Lipoprotein Combine Index (LCI), Triglyceride-Glucose (TyG), TyG-BMI, TyG-WC, and Thrombolysis In Myocardial Infarction (TIMI) risk index), and hepatic indices (Hepatic Steatosis Index (HSI) and ALD/NAFLD index (ANI)) were calculated and compared between the groups.

**Results:**

WHR,VAI, AIP, LAP, CMI, LCI, TyG, and TIMI risk index values were significantly higher in the MUNO phenotype compared to the MHO phenotype (WHR: 0.97 vs. 0.95; VAI: 3.16 vs. 1.33; AIP: 0.58 vs. 0.25; LAP: 78.87 vs. 55.79; CMI: 2.69 vs. 1.25; LCI: 27.91 vs. 12.11; TyG: 9.21 vs. 8.41; TIMI: 18.66 vs. 15.63; p < 0.001). The highest and lowest HSI and ANI values were detected in the MUO phenotype. After adjustment for age, sex, physical activity, and years of education, VAI showed the highest Odds Ratio for MUNO (OR: 5.65; 95% CI: 5.12, 6.24) and MUO (OR: 5.40; 95% CI: 5.89, 5.95) compared to the MHNO phenotypes (p < 0.001). The ANI indices was associated with a reduced risk of MUO (OR: 0.76; 95% CI: 0.75–0.78), MUNO (OR: 0.88; 95% CI: 0.87–0.90), and MHO (OR: 0.79; 95% CI: 0.77–0.81) phenotypes (p < 0.001).

**Conclusion:**

MUNO phenotype was exposed to a higher risk of cardiovascular disease compared to the MHO phenotype. VAI was found to be the optimal index for cardiovascular risk assessment.

**Supplementary Information:**

The online version contains supplementary material available at 10.1186/s12902-023-01372-9.

## Background

The prevalence of obesity is increasing rapidly worldwide, causing susceptibility to pro-inflammatory state by increasing inflammatory mediators, such as Tumor Necrosis Factor-alpha (TNFα), and Interleukin-6 (IL-6), and reducing the level of adiponectin. The subsequent oxidative stress and inflammation lead to insulin resistance, atherosclerosis, cardiovascular events, Hypertension, diabetes mellitus, metabolic syndrome, and cancer [1, 2]. The Plasma increased levels of liver enzymes, including Aspartate Aminotransferase (AST), Alanine Transaminase (ALT), and Gamma-Glutamyl Transferase (GGT)), beside the Fatty Liver Index (FTI) as a simple and accurate predictor of hepatic steatosis, are more commonly reported in at-risk obese individuals [3].

Obesity can be either metabolically healthy (normal) or metabolically unhealthy (abnormal) [4]. Approximately 20% of patients with Metabolically Healthy Normal Weight (MHNW) lose their normal metabolic status after 10.9 years, despite normal tissue sensitivity to insulin and reduced risk of cardio-metabolic diseases [5].

The Metabolically Healthy Obese (MHO) phenotype include a subgroup of obese individuals with a favorable metabolic profiles, characterized by normal insulin sensitivity, relative fat mass reduction, proper adipose tissue function, a normal lipid profile, lack of Hypertension, a favorable immune profile, normal inflammatory levels, and a hormonal profile. Evidence suggests that the risk of Cardiovascular Diseases (CVD), Type 2 Diabetes Mellitus (T2DM), and mortality in this phenotype is not higher than that of non-obese individuals [6–8]. However, in some studies, the increased risk of T2DM progression, hypertriglyceridemia, reduction of High-Density Lipoprotein-Cholesterol (HDL-C) level, CVD, cerebrovascular events, and metabolic disorders has been reported. The risk of CVD in this phenotype is worse than the risk in the MHNW, and better than the risk in the Metabolically Unhealthy Normal Weight (MUNW) phenotypes [5, 9, 10]. Approximately half of the people with this phenotype progress toward the Metabolically Unhealthy Obese (MUO) phenotype over 10.9 years [5, 11]. Overall, MHO phenotype comprises about 30% of the obese population [12].

Moreover, the MUNW phenotypes accounts for nearly 30% of the adult population with normal weights [13] with an estimated global prevalence of 5–45%. The incidence of this phenotype significantly increases in the population aged ≥ 50 years. It is also more prevalent in women than men and has a positive relationship with age [4]. Previous research suggests the triple risk of CVD in this phenotype [14]. Additionally, increased insulin resistance, inflammation, Triglyceride (TG) level, blood pressure, and Fasting Plasma Glucose (FPG), beside decreased HDL-C levels, have been reported in this population [4].

On the other hand, the subgroup of obese individuals with the MUO phenotype and metabolic syndrome are different regarding Systolic (SBP), Diastolic Blood Pressure (DBP), FPG, HDL-c, TG level, Body Mass Index (BMI), and Waist Circumference (WC) in some populations. Previous studies have reported an increase in insulin resistance following deterioration of metabolic status [15].

The ObAGE is in an excellent position to approach aging as a process whose expression involves multiple factors from the early stages of a person’s life; understand how longitudinal changes in health trajectories impact the biological mechanisms of aging; identify potential resilience mechanisms that help prevent unhealthy aging [16].

There are four main epigenetic mechanisms, including DNA methylation, histone modification, chromatin remodeling, and noncoding RNA (ncRNA), which exert different effects on metabolic diseases. Genetic and non-genetic factors, including ageing, diet, and exercise, interact with epigenetics and jointly affect the formation of a phenotype. Understanding epigenetics could be applied to diagnosing and treating metabolic diseases in the clinic, including epigenetic biomarkers, epigenetic drugs, and epigenetic editing [17].

Therefore, the present study aimed to determine the frequency of metabolic obesity phenotypes, to compare anthropometric indices, lipid profiles, liver enzymes, FPG level, and blood pressure between these phenotypes, and to determine the association of liver function and cardiovascular function indices with metabolically obesity phenotypes.

## Materials and methods

### Research design and participants

**Fig. 1:**
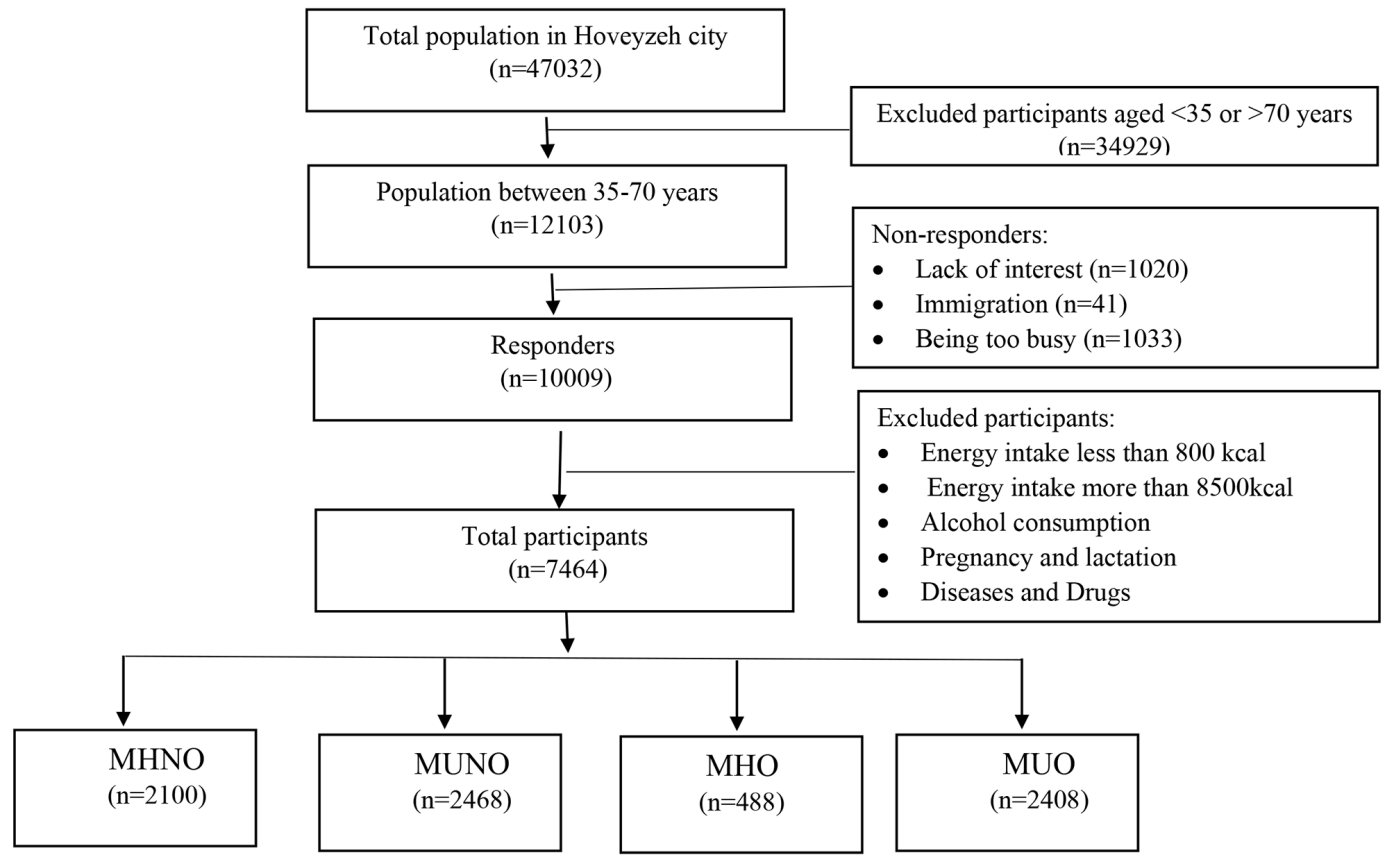
Flow chart of participant selection

This cross-sectional study was conducted on the initial phase data of the Hoveyzeh Cohort Study, a Prospective Epidemiological Research Study in Iran (the PERSIAN Cohort), and a population-based research on non-communicable diseases in an Arab community in the Southwest of Iran [18, 19]. All eligible 35-70-year-old individuals in the study area were selected by the census sampling method and invited to participate in the enrollment phase. The inclusion criteria were willingness to participate in the study and being in the age range of 35–70 years. According to Fig. 1, out of the total population of Hoveyzeh (47,032), 34,929 people aged ˂ 35 or ˃ 70 years left the study, while 12,103 individuals in the age range of 35–70 years remained in the study. After excluding non-responders due to a lack of interest (n = 1020), immigration (n = 41), or being overly busy (n = 1033), a total of 10,009 people were included in this study; some of the participants were members of the same family.

Then, patients with aged 35–70 years, who met the following criteria, were excluded from the study: energy intake of ˂800 kcal or ˃8500 kcal; pregnant or lactating women; incomplete demographic, anthropometric, or biochemical data; bariatric surgery in the last year; adherence to a special diet; inflammatory disease; liver, kidney, adrenal, thyroid, or heart disease; acute diseases; cancer; alcohol consumption; and use of specific medicines. Finally, the study was performed on 7,464 participants (female: 4605; male: 2859), including 1,774 people with normal weight, 2,792 overweight individuals, and 2895 people with obesity.

Healthy and unhealthy metabolic phenotypes were defined based on The National Cholesterol Education Program (NCEP) and Adult Treatment Panel (ATP) III criteria: (1) WC ≥ 102 cm in males and WC ≥ 88 cm for females; (2) FPG ≥ 100 mg/dl or receiving antidiabetic therapy for T2DM; (3) Serum TG ≥ 150 mg/dl; (4) HDL-C ≤ 40 mg/dl in males and ≤ 50 mg/dl in females or receiving anti-dyslipidemia therapy; and (5) SBP ≥ 130 mmHg or DBP ≥ 85 mmHg or receiving anti-hypertensive therapy. Individuals with a healthy phenotype meet none or only one of the NCEP ATP III criteria, whereas individuals with an unhealthy phenotype meet two or more than of these criteria [20].

Based on this, participants were classified into four groups: (1) MHNO group; Metabolically Healthy Normal Weight-Overweight (Non-Obese) individuals with none or only one of the NECP ATP III criteria and BMI = 18.5–29.9 kg/m^2^; (2) MUNO group; Metabolically Unhealthy Normal weight and Overweight (Non-Obese) individuals with two or more than two NECP criteria and BMI = 18.5–29.9 kg/m^2^; (3) MHO group; Metabolically Healthy Obese people with none or only one of the NCEP ATP III criteria and BMI ≥ 30 kg/m^2^; (4) MUO group; Metabolically Unhealthy Obese people with two or more than two of the NCEP ATP III criteria and BMI ≥ 30 kg/m^2^ (Fig. 1). Regarding the metabolic conditions, the metabolically healthy status is better than metabolically unhealthy status.

### Definition

Obesity was defined as BMI ≥ 30 kg/m^2^ according to the World Health Organization (WHO) definition. Also, DM was defined as FPG ≥ 126 mg/dl. Patients who were treated with glucose-lowering medicines were identified as diabetic. Dyslipidemia was defined as serum TG ≥ 150 mg/dl, or HDL-c ≤ 40 mg/dl in males and ≤ 50 mg/dl in females, or both. Additionally, physical activity was investigated using a questionnaire, such as occupational, free time, and domestic activities, nutrition, and sleep, and expressed as the Metabolic Equivalent of Tasks (MET, hours/weekly) [21].

### Ethical approval

This study was performed according to the ethical principles of the Declaration of Helsinki. Approval to undertake the study was granted by the Ethics Committee of Ahvaz Jundishapur University of Medical Sciences, Ahvaz, Iran (Date: August 4, 2021; No: IR.AGUMS.HGOLESTAN.REC.1400.109). Written informed consent was obtained from all the participants.

### Anthropometric and blood pressure measurements

Body weight (kg), was measured after overnight fasting using a vertical scale (Seca 755), while height (cm) was measured without shoes using a stadiometer (Seca 206). The BMI was also calculated as body weight (kg), divided by the square of height (m). Additionally, WC (cm), Wrist Circumferences (WrC, cm), and Hip Circumference (HC, cm) were measured using Seca non-stretchable tapes. The Waist to Hip Ratio (WHR) and Waist to Height Ratio (WHtR) were calculated by dividing WC by HC and Height (cm), respectively. Other Anthropometric indices were calculated based on the following formulae [22, 23]:

1. Weight adjusted Waist Index (WWI): WC (cm) divided by the square root of weight (kg) (cm/√kg);
2. Body Adiposity Index (BAI): [HC (cm) / height (M) × 1.5] – 18;
3. Visceral Adiposity Index (VAI) in males: [WC/ (39.68 + 1.88 × BMI)] × (TG (mmol/L)/1.03) × (1.31/ HDL-c (mmol/L);
4. VAI in females: [WC/ (36.58 + 1.89 × BMI)] × (TG/0.81) × (1.52/HDL-c);

Moreover, the SBP, DBP, and heart rate of the participants were measured. To evaluate blood pressure, they were asked to sit on a chair with no physical activity for one hour.

### Biochemical assessments

A 10-cc venous blood sample was taken from all participants after 12 h of overnight fasting. The blood samples were centrifuged at 300 rpm for 10 min, and the sera were stored at -70 °C. The Complete Blood Count (CBC) was calculated using an automated hematology analyzer (Nihon Kohden 6510-k, Japan). Total Cholesterol (TC), TG, HDL-C, and serum glucose levels were also measured using a commercial kit (Pars Azmoon, Tehran, Iran). The Serum level of low-density lipoprotein-cholesterol (LDL-c) was calculated according to the Friedewald equation. Finally, ALT, AST, and Alkaline Phosphatase (ALP) were measured using GPT/ALAT & GOT/AST commercial kits (Pars Azmoon, Tehran, Iran).

### Cardiovascular and hepatic index measurements

The cardiovascular and hepatic indices, including the Atherogenic Index of Plasma (AIP), Lipid Accumulation Product (LAP), Cardio-Metabolic Index (CMI), Lipoprotein Combine Index (LCI), Alcoholic liver Disease (ALD)/Non-Alcoholic Fatty Liver Disease (NAFLD) Index (ANI), and Hepatic Steatosis Index (HSI), were calculated based on biochemical parameters, using the following formulae [24, 25].

***Hepatic index***:

1. HSI: 8 × (ALT/AST ratio) + BMI (+ 2, if female; +2, if diabetes mellitus);
2. ANI: -58.5 + 0.637 (Mean Corpuscular Volume (MCV)) + 3.91 (AST/ALT) – 0.406 (BMI) + 6.35 (for male gender);

***Cardiovascular index***:

1. LAP (in men): [WC–65] × [TG].
2. LAP (in women): [WC–58] × [TG].
3. AIP: Log (TG/ HDL-C).
4. CMI: TG/HDL-C × (Waist-to-Height Ratio).
5. The Thrombolysis in Myocardial Infarction (TIMI) risk index: heart rate (bpm) × (Age/10)2 /SBP (mmHg).
6. The TyG index: Ln [TG (mg/dl) × FPG (mg/dl)/2]
7. TyG-BMI: TyG index × BMI.
8. TyG-WC: TyG index × WC (cm).
9. LCI: TC×TG×LDL/HDL-C.

### Statistical analysis

To compare quantitative variables, Analysis of Variance (ANOVA) test was performed. Chi-square test was also used to compare qualitative variables between groups. Linear regression analysis was performed to evaluate the association of hepatic index and cardiovascular indices with quantitative variables. Moreover, a Logistic regression test was conducted to calculate the Odds Ratio (OR) and 95% Confidence Intervals (95% CI). All data were expressed as mean ± Standard Deviation (± SD) and frequency (%), and analyzed using IBM SPSS Version 24 (IBM SPSS statistics, Armonk, USA). Finally, the normal distribution of variables was examined using the Kolmogorov-Smirnov test. A P-value less than 0.05 was considered statistically significant.

## Results

The baseline characteristics of the study populations are presented in Table 1. The mean (SD) age of the participants was 49.0 (9.25) years, and 38.3% of them were male. 19.6% of the participants were smokers, and the majority of them were married (87.1%). Overall, 488 (6.54%) participants were MHO, 2,408 (32.26%) were MUO, 2,468 (33.06%) were MUNO, and 2,100 (28.14%) were MHNO phenotype. The highest and lowest levels of physical activity were reported in the MHNO and MUO phenotypes, respectively, while the highest and lowest frequencies of diabetes and dyslipidemia were attributed to the MUNO and MHO phenotypes, respectively. The highest frequencies of Ischemic Heart Diseases (IHD) and Myocardial Infarction (MI) were shown in the MUO phenotype, and the highest frequency of stroke was shown in the MUNO phenotype. Besides, the mean weight, height, WC, HC, WrC, blood pressure, and heart rate were significantly different between obesity phenotypes (Table 1).

**Table 1:** Baseline characteristics according to the metabolic obesity phenotypes in a cross-sectional study of Hoveyzeh Cohort

Variables	Total (N = 7464)	MHNO (N = 2100)	MUNO (N = 2468)	MHO (N = 488)	MUO (N = 2408)	P-value^*^
Age (years)	49.00 ± 9.25	47.60 ± 9.31	51.34 ± 9.69	45.51 ± 8.49	48.54 ± 8.49	< 0.001
Gender, male n (%)	2859 (38.3)	927 (44.1)	1182 (47.9)	106 (21.7)	644 (26.7)	< 0.001
Marital state n (%)						
Single	270 (3.6)	111 (5.3)	75 (3.0)	19 (3.9)	65 (2.7)	< 0.001
Married	6500 (87.1)	1834 (87.3)	2127 (86.2)	436 (89.3)	2103 (87.3)	˂0.001
Widow	561 (7.5)	117 (5.6)	225 (9.1)	23 (4.7)	196 (8.1)	˂0.001
Divorced	133 (1.8)	38 (1.8)	41 (1.7)	10 (2.0)	44 (1.8)	˂0.001
Current smoking, n (%)	1465 (19.6)	472 (22.5)	620 (25.1)	39 (8.0)	334 (13.9)	< 0.001
Residence Type (Urban/rural)	4725/2739	1140/960	1577/891	342/146	1666/742	˂0.001
Education (years)	4.03 ± 4.98	4.18 ± 5.17	4.12 ± 5.07	4.50 ± 4.85	3.72 ± 4.23	0.001
Weight (kg)	77.68 ± 14.87	66.19 ± 10.20 ^a, b, c^	73.28 ± 10.52 ^d, e^	87.69 ± 12.09 ^f^	90.17 ± 11.89	< 0.001
Height (cm)	164.13 ± 8.98	164.59 ± 9.25 ^a, b, c^	165.76 ± 9.12 ^d, e^	161.40 ± 8.50 ^f^	162.62 ± 8.30	< 0.001
Waist circumference (cm)	99.7 ± 11.76	88.73 ± 7.62 ^a, b, c^	97.12 ± 7.06 ^d, e^	107.14 ± 9.55 ^f^	110.41 ± 8.40	< 0.001
Hip circumference (cm)	104.10 ± 9.75	97.09 ± 6.51 ^a, b, c^	99.88 ± 5.78 ^d, e^	113.16 ± 7.49	112.72 ± 7.76	< 0.001
Wrist circumference (cm)	17.42 ± 1.31	16.67 ± 1.03 ^a, b, c^	17.15 ± 1.08 ^d, e^	17.88 ± 1.16 ^f^	18.26 ± 1.28	< 0.001
Systolic blood pressure	113.11 ± 18.36	106.25 ± 13.61 ^a, c^	116.91 ± 20.15 ^d^	107.83 ± 12.11 ^f^	116.27 ± 19.14	< 0.001
Diastolic blood pressure	71.38 ± 11.15	67.60 ± 9.24 ^a, b, c^	73.15 ± 11.77 ^d^	69.35 ± 8.81 ^f^	73.25 ± 11.54	< 0.001
Heart rate	78.63 ± 9.58	77.02 ± 9.24 ^a, b, c^	79.09 ± 10.05	78.38 ± 8.30 ^f^	79.60 ± 9.45	< 0.001
Physical activity (MET)	36.90 ± 5.48	37.86 ± 5.98 ^a, c^	36.52 ± 5.52 ^d^	37.41 ± 4.90 ^f^	36.35 ± 4.98	< 0.001
Diabetes, n (%)	1650 (22.1)	158 (7.5)	778 (31.5)	14 (2.9)	700 (29.1)	< 0.001
Dyslipidemia, n (%)	3227 (43.2)	425 (20.2)	1460 (59.2)	77 (15.8)	1265 (52.5)	< 0.001
Ischemic heart diseases, n (%)	986 (13.2)	188 (9)	356 (14.4)	55 (11.3)	387 (16.1)	< 0.001
Myocardial infarction, n (%)	129 (1.72)	18 (0.86)	53 (2.15)	5 (1.02)	53 (2.20)	0.001
Stroke, n (%)	124 (1.66)	17 (0.80)	52 (2.11)	5 (1.00)	50 (2.07)	0.001

Data are means ± SD for quantitative variables and frequency (percent) for qualitative variables. MET: metabolic activity of task

^*^ ANOVA for quantitative variables, chi-square for qualitative variables

^a^ Significant difference between MHNW compared to MUNW.

^b^ Significant difference between MHNW compared to MHO.

^c^ Significant difference between MHNW compared to MUO.

^d^ Significant difference between MUNW compared to MHO.

^e^ Significant difference between MUNW compared to MUO.

^f^ Significant difference between MHO compared to MUO.

The highest mean levels of FBS, TG, Cholesterol, and ALP were found in the MUNO phenotype, and the highest mean level of ALT was found in the MUO phenotype. The highest mean values of in WHR, WHtR, BMI, and WWI were attributed to the MUO phenotypes. The WHR and VAI were higher in the MUNO phenotype compared to the MHO phenotypes. Also, the highest mean values of BAI and VAI were found in the MHO and MUNO phenotypes, respectively (Table 2).

**Table 2:** Anthropometric and biochemical measures according to the metabolic obesity phenotypes in a cross sectional study of Hoveyzeh Cohort

Variables	Total (N = 7464)	MHNO (N = 2100)	MUNO (N = 2468)	MHO (N = 488)	MUO (N = 2408)	P-Value^*^
Fasting blood sugar (mg/dl)	112.61± 49.50	94.72 ± 28.57 ^a, b, c^	125.51 ± 60.30 ^d, e^	89.74 ± 6.80 ^f^	119.64 ± 50.43	< 0.001
Triglycerides (mg/dl)	162.19 ± 105.70	109.67 ± 51.79 ^a, c^	197.65 ± 131.37 ^d, e^	104.27 ± 29.38 ^f^	183.39 ± 98.06	< 0.001
Total Cholesterol (mg/dl)	189.05 ± 40.44	182.25 ± 36.84 ^a, b, c^	192.36 ± 43.49	188.59 ± 31.04	191.68 ± 41.16	< 0.001
High-density lipoprotein (mg/dl)	50.15 ± 11.86	54.89 ± 11.91 ^a, b, c^	46.63 ± 11.01 ^d, e^	57.51 ± 10.15 ^f^	48.12 ± 10.96	< 0.001
Low-density lipoprotein (mg/dl)	106.75 ± 33.03	105.40 ± 31.48 ^b^	106.95 ± 34.82	110.06 ± 27.56	107.04 ± 33.46	0.034
Aspartate transaminase (U/L)	18.52 ± 9.46	18.47 ± 7.82	18.61 ± 11.07	17.55 ± 6.68	18.67 ± 9.44	0.110
Alanine aminotransferase (U/L)	21.00 ± 14.45	18.72 ± 12.94 ^a, c^	22.03 ± 15.30 ^d^	19.44 ± 13.78 ^f^	22.26 ± 14.69	< 0.001
Alkaline phosphatase (U/L)	211.58 ± 61.81	199.37 ± 59.25 ^a, c^	219.29 ± 62.41 ^d^	199.71 ± 59.59 ^f^	216.74 ± 61.89	< 0.001
Waist-To-Hip Ratio	0.96 ± 0.07	0.91 ± 0.06 ^a, b, c^	0.97 ± 0.06 ^d, e^	0.95 ± 0.07 ^f^	0.98 ± 0.06	< 0.001
Waist-To-Height Ratio	0.61 ± 0.08	0.54 ± 0.05 ^a, b, c^	0.59 ± 0.05 ^d, e^	0.66 ± 0.06 ^f^	0.68 ± 0.06	< 0.001
Body mass index	28.84 ± 5.14	24.39 ± 2.91 ^a, b, c^	26.59 ± 2.45 ^d, e^	33.58 ± 2.99 ^f^	34.06 ± 3.50	< 0.001
Body Adiposity Index	31.80 ± 6.33	28.24 ± 4.88 ^a, b, c^	29.02 ± 4.27 ^d, e^	37.42 ± 5.04 ^f^	36.62 ± 5.56	< 0.001
Visceral Adiposity Index	2.47 ± 2.04	1.40 ± 0.70 ^a, c^	3.16 ± 2.53 ^d, e^	1.33 ± 0.43 ^f^	2.93 ± 1.96	< 0.001
Weight-adjusted Waist Index	11.37 ± 0.84	10.95 ± 0.81 ^a, b, c^	11.40 ± 0.82 ^e^	11.48 ± 0.78 ^f^	11.67 ± 0.74	< 0.001

Data are means ± SD for quantitative variables

^*^ ANOVA for quantitative variables

^a^ Significant difference between MHNW compared to MUNW.

^b^ Significant difference between MHNW compared to MHO.

^c^ Significant difference between MHNW compared to MUO.

^d^ Significant difference between MUNW compared to MHO.

^e^ Significant difference between MUNW compared to MUO.

^f^ Significant difference between MHO compared to MUO.

**Fig. 2:**
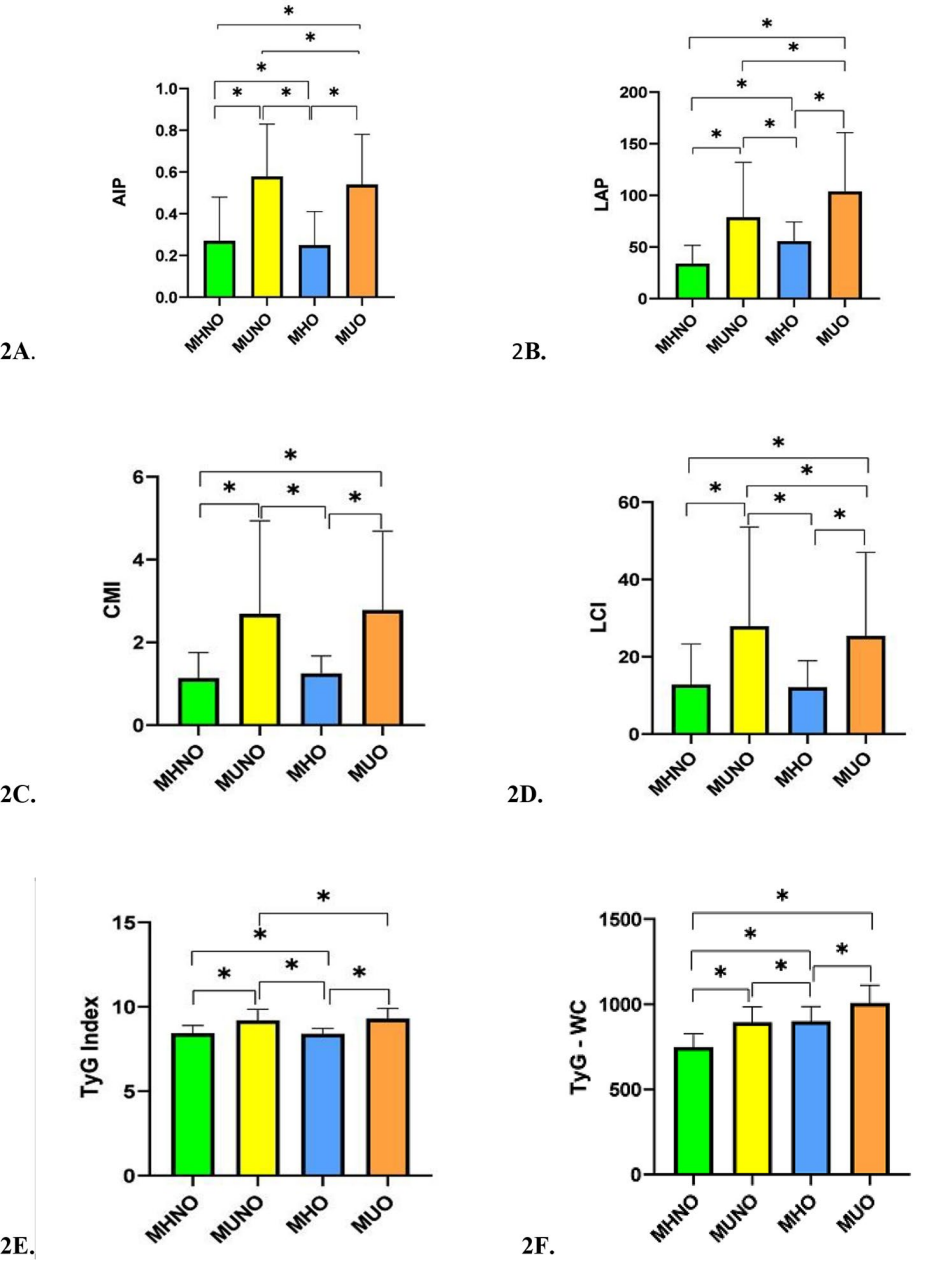
Cardio-metabolic indices (2 A: AIP; 2B: LAP; 2 C: CMI; 2D: LCI; 2E: TyG Index; 2 F: TyG - WC) according to metabolic obesity phenotypes in a cross sectional study of Hoveyzeh Cohort Study

**Fig. 3:**
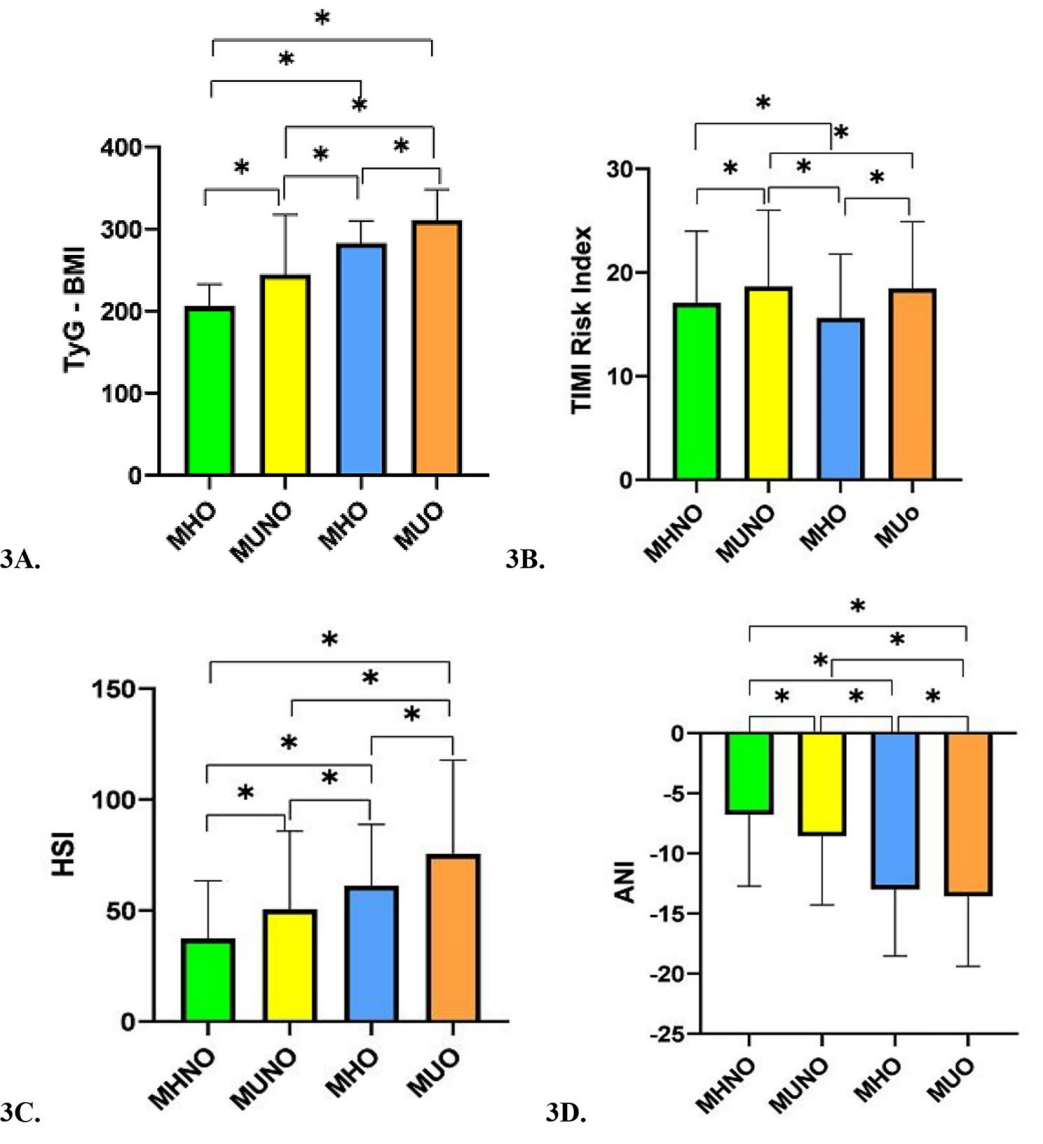
Cardio-metabolic (3 A: TyG – BMI; 3B: TIMI risk index) and Hepatic indices (3 C: HIS; 3D: ANI) according to metabolic obesity phenotypes in a cross sectional study of Hoveyzeh Cohort

The highest mean values of LAP, CMI, TyG-Index, TyG-BMI, and TyG-WC were observed in the MUO phenotype, and the highest mean values of AIP, LCI, and TIMI risk index were attributed to the MUNO phenotype. Based on the results, AIP, LAP, CMI, LCI, TyG index, and TIMI risk index were significantly higher in the MUNO phenotype compared to the MHO phenotypes (Fig. 2A F &, Fig. 3A and B). The highest HSI, and lowest ANI values were found in the MUO phenotype (Fig. 3C and D).

According to Spearman’s correlation coefficient, VAI consistently had the most significant correlation with various cardio-metabolic variables (AIP, LAP, CMI, LCI, and TyG Index). However, BAI had the most significant correlation with hepatic risk factors variables (HSI and ANI) (Supplementary file).

The multivariate-adjusted ORs (95% CI) for the metabolic obesity phenotypes, based on the risk comparison of MUNO, MHO, and MUO phenotypes with MHNO as the reference, are presented in Tables 3 and 4 (model 2: after adjustment for age, gender, physical activity, and years of education). Among anthropometric, cardio-metabolic, and hepatic indices, VAI had the highest multivariate-adjusted OR for metabolic obesity phenotypes based on the comparison of MUNO and MUO phenotypes with MHNO (OR = 5.65, 95% CI: 5.12–6.24 and OR = 5.40, 95% CI: 5.89–5.95, respectively) (p < 0.0001). The association of TIMI risk index with the MUO, MUNO, and MHO phenotypes was weak and non-significant. Moreover, the results showed that the ANI indices was associated with a reduced risk of MUO, MUNO, and MHO phenotypes. The highest ORs for cardio-metabolic, hepatic, and anthropometric indices were observed in the MUO phenotype.

**Table 3:** Multivariable logistic regression analysis on anthropometric measures between metabolically obesity phenotypes in a cross sectional study of Hoveyzeh Cohort

Variables	MHNO	MUNO		MHO		MUO	
		OR (95%CI)	P-V	OR (95%CI)	P-V	OR (95%CI)	P-V
**Waist-To-Hip Ratio**	Reference						
Model 1		1.17 (1.16–1.18)	< 0.0001	1.09(1.08–1.11)	< 0.0001	1.20 (1.18–1.21)	< 0.0001
Model 2		1.18 (1.16–1.19)	< 0.0001	1.16 (1.14–1.18)	< 0.0001	1.24 (1.23–1.26)	< 0.0001
**Waist-To-Height Ratio**	Reference						
Model 1		1.18 (1.17–1.20)	< 0.0001	1.56 (1.52–1.59)	< 0.0001	1.63 (1.60–1.66)	< 0.0001
Model 2		1.26 (1.24–1.28)	< 0.0001	1.97 (1.90–2.04)	< 0.0001	2.07 (2.00-2.13)	< 0.0001
**Body Adiposity Index**	Reference						
Model 1 Model 2		1.04 (1.02–1.05) 1.13 (1.11–1.15)	0.0001 0.0001	1.44 (1.41–1.47) 2.09 (2.01–2.18)	< 0.0001 < 0.0001	1.40 (1.38–1.42) 2.03 (1.96–2.10)	< 0.0001 < 0.0001
**Visceral Adiposity Index**	Reference						
Model 1		5.54 (5.03–6.10)	< 0.0001	0.81 (0.68–0.97)	0.019	5.28 (4.80–5.82)	< 0.0001
Model 2		5.65 (5.12–6.24)	< 0.0001	0.84 (0.71-1.00)	0.055	5.40 (4.89–5.95)	< 0.0001
**Weight-adjusted Waist Index**	Reference						
Model 1		2.08 (1.93–2.25)	< 0.0001	2.34 (2.06–2.66)	< 0.0001	3.18 (2.92–3.45)	< 0.0001
Model 2		3.29 (2.93–3.70)	< 0.0001	3.48 (2.89–4.18)	< 0.0001	5.20 (4.61–5.87)	< 0.0001

Model 1: unadjusted

Model 2: Adjusted for age, gender, physical activity, education years

**Table 4:** Multivariable logistic regression analysis on cardio-metabolic and hepatic indices between metabolically obesity phenotypes in a cross sectional study of Hoveyzeh Cohort;

Variables	MHNO	MUNO		MHO		MUO	
		OR (95%CI)	P-V	OR (95%CI)	P-V	OR (95%CI)	P-V
**AIP**	Reference						
Model 1		1.87 (1.81–1.94)	< 0.001	0.94 (0.89–0.98)	0.010	1.76 (1.70–1.82)	< 0.001
Model 2		2.02 (1.94–2.10)	< 0.001	1.01 (0.96–1.06)	0.811	1.96 (1.89–2.04)	< 0.001
**LAP**	Reference						
Model 1		1.08 (1.08–1.09)	< 0.001	1.06 (1.05–1.06)	< 0.001	1.09 (1.09–1.10)	< 0.001
Model 2		1.08 (1.08–1.09)	< 0.001	1.06 (1.05–1.06)	< 0.001	1.09 (1.09–1.10)	< 0.001
**CMI**	Reference						
Model 1		1.24 (1.23–1.26)	< 0.001	1.04 (1.02–1.06)	< 0.001	1.25 (1.23–1.26)	< 0.001
Model 2		1.26 (1.25–1.28)	< 0.001	1.06 (1.04–1.08)	< 0.001	1.27 (1.25–1.28)	< 0.001
**LCI**	Reference						
Model 1		1.09 (1.08–1.09)	< 0.001	0.99 (0.98-1.00)	0.081	1.08 (1.07–1.09)	< 0.001
Model 2		1.09 (1.08–1.10)	< 0.001	1.00 (0.99–1.01)	0.961	1.09 (1.08–1.09)	< 0.001
**TyG Index**	Reference						
Model 1		1.37 (1.35–1.39)	< 0.001	0.98 (0.95-1.00)	0.049	1.34 (1.32–1.36)	< 0.001
Model 2		1.38 (1.36–1.41)	< 0.001	1.01 (0.98–1.03)	0.761	1.37 (1.35–1.40)	< 0.001
**TyG-BMI**	Reference						
Model 1		1.05 (1.05–1.06)	< 0.001	1.12 (1.11–1.12)	< 0.001	1.15 (1.15–1.16)	< 0.001
Model 2		1.06 (1.05–1.06)	< 0.001	1.13 (1.12–1.13)	< 0.001	1.16 (1.15–1.17)	< 0.001
**TyG-WC**	Reference						
Model 1		1.02 (1.02–1.02)	< 0.001	1.02 (1.02–1.03)	< 0.001	1.04 (1.03–1.04)	< 0.001
Model 2		1.02 (1.02–1.02)	< 0.001	1.03 (1.02–1.03)	< 0.001	1.04 (1.04–1.04)	< 0.001
**TIMI**	Reference						
Model 1		1.03 (1.02–1.04)	< 0.001	0.96 (0.95–0.98)	< 0.001	1.00 (0.99-1.00)	0.232
Model 2		0.93 (0.92–30.95)	< 0.001	0.96 (0.93–0.99)	0.017	0.91 (0.89–0.92)	< 0.001
**ANI**	Reference						
Model 1		0.94 (0.93–0.95)	< 0.001	0.83 (0.81–0.84)	< 0.001	0.81 (0.80–0.82)	< 0.001
Model 2		0.88 (0.87–0.90)	< 0.001	0.79 (0.77–0.81)	< 0.001	0.76 (0.75–0.78)	< 0.001
**HIS**	Reference						
Model 1		1.01 (1.01–1.01)	< 0.001	1.02 (1.02–1.02)	< 0.001	1.03 (1.03–1.03)	< 0.001
Model 2		1.10 (1.09–1.11)	< 0.001	1.09 (1.09–1.11)	< 0.001	1.12 (1.11–1.13)	< 0.001

Model 1: unadjusted; Model 2: Adjusted for age, gender, physical activity, education years

## Discussion

In this study, the highest and lowest frequencies of phenotypes were attributed to the MUNO (33.06%) and MHO (6.54%) phenotypes, respectively. In previous studies, the prevalence of MHO phenotype was estimated at 6–38% among obese individuals, and the prevalence of the MUNW phenotype ranged from 5 to 45% [4, 26]. The observed variations were attributed to differences in age, sex, geographical location of residence, and definitions of healthy and unhealthy metabolic status.

In the present study, the mean values of WC, HC, WrC, WHR, WHtR, and WWI were significantly different between obesity phenotypes. They were statistically higher in metabolically unhealthy phenotypes compared to metabolically healthy phenotypes, in all BMI categories. They were also higher in the non-obese phenotypes as compared to obese phenotypes. Therefore, unhealthy phenotypes were mostly related to visceral fat, insulin resistance, and increased cardiovascular risk [15, 23]. In previous studies, WC and WHR were higher in the MHO and MUO phenotypes compared to the MHNW phenotype, while they were lower in the MHO phenotype as compared to the MUO phenotype [27]; these studies are dissimilar to our research in classifying the participants based on BMI to define obesity and non-obesity. The Neck Circumference (NC), HC, and WrC are recognized as simple and practical indices of body fat distribution [28]. Generally, NC is an acceptable predictor of a higher BMI. Besides, WrC which is as important as NC, is a suitable indicator of metabolic status. Individuals with metabolic syndrome have a higher WrC compared to normal people. In the current study on individuals aged 35–70 years, the highest WC and WrC were found in the MUO phenotype. Therefore, this indicators are superior to BMI for obesity phenotype and CVD in children and adolescents [29–31]. Moreover, WHtR is helpful in determining abdominal obesity [32]. It has been previously reported that 7-18-years-old students with general and abdominal obesity have higher NC, HC, and WrC values compared to those with only abdominal obesity. The corresponding values were higher in obese adolescents with metabolic syndrome compared to other phenotypes and also higher in non-obese adolescents with metabolic syndrome compared to the MHO phenotype. Overall, there was a strong correlation between these indicators and metabolic syndrome among students. In these studies, NC, HC, and WrC identified as valuable, low-cost, and accessible indicators for identifying the MUNO phenotypes and helped prevent serious disorders, such as CVD [33].

In the present study, the levels of ALT and ALK were higher in unhealthy phenotypes as compared to healthy phenotypes. The maximum HSI and minimum ANI values were observed in the MUO phenotype. According to the results, an increase in the ANI index was associated with a decline in the risk of MUO, MUNO, and MHO phenotypes compared to MHNO phenotype. Higher plasma levels of liver enzymes were associated with metabolic syndrome, insulin resistance, and NAFLD, leading to T2DM and CVD [34]. Evidence suggests that the levels of AST, ALT, and GGT are significantly lower in women with the MHO phenotype compared to at-risk women for obesity; lower levels of liver enzymes, body fat content, and insulin resistance can contribute to a favorable metabolic profile in the MHO phenotype, despite a high body fat content [3, 35]. An increase in HSI suggests hepatic steatosis, which is more common in obese and metabolically unhealthy individuals. It is known that metabolic abnormalities associated with obesity reinforce the risk of NAFLD. Meanwhile, obesity increases the risk of NAFLD independently. In our study, the ANI value was lower in obesity and metabolically unhealthy phenotypes, which might indicate increased risk of NAFLD in the MUO and MHO phenotypes compared to non-obese individuals. In previous studies, the risk of NAFLD progression increased in the MHO and MUNW phenotypes compared to the MHNW phenotype. Also, the risk of NAFLD was significantly higher in the MUO phenotype compared to MHO phenotype [36]. It has been shown that ANI helps distinguish ALD from NAFLD with high accuracy [24]. In obesity, oxidative stress, along with the release of pro-inflammatory cytokines, such as leptin, IL-6, and TNF-α from adipose tissue, can lead to NAFLD, non-alcoholic steatohepatitis, fibrosis, and liver cirrhosis progression [2, 37]. Lifestyle changes in non-obese individuals with NAFLD significantly improve the grade of hepatic steatosis [38]. Duo to the increased incidence of NAFLD following the increased frequency of metabolic abnormalities, consultation with experts is strongly needed for management of patients, regardless of BMI. Treatment planning is also essential to change abnormal weight and unhealthy metabolic status. Moreover, it is advantageous to use hepatic indices to identify steatosis and differentiate NAFLD from ALD, especially in metabolically unhealthy phenotype [36].

The better metabolic status of the MHO phenotype compared to metabolically unhealthy Lean phenotype could be related to body fat content; therefore, body fat is superior to BMI as a cardiovascular risk predictor [39, 40]. In our study, the BAI value was higher in obese phenotypes (MHO and MUO) compared to the non-obese phenotypes (MHNO and MUNO). The MHO phenotype, with highest BAI was mainly associated with NAFLD and steatosis compared to the MUNO phenotype. Also, the BAI value was higher in the MUNO phenotype compared to the MHNO phenotype. Research indicate that a normal BMI and a high body fat content are more significantly associated with metabolic syndrome [41].

In our study, the highest mean values of VAI, AIP, LCI, and TIMI were observed in the MUNO phenotype. However, the highest mean values of LAP and TyG-BMI were attributed to the MUO phenotype. Based on the results, the values of VAI, AIP, LAP, CMI, LCI, TyG Index, and TIMI risk index were higher in metabolically unhealthy people compared to the healthy phenotype. The VAI had the highest OR for the MUNO and MUO phenotypes compared to MHNO after adjustment for confounding factors. This index increased the risk of MUNO phenotype by 5.65 times and the risk of MUO phenotype by 5.4 times compared to MHNO phenotype. An increase in the AIP index elevated the risk of MUNO phenotype by 2.02 times and the risk of MUO phenotype by 1.96 times compared to the MHNO phenotype. In previous studies, individuals with higher AIP and VAI values had more Hypertension, dyslipidemia, metabolic syndrome, and CVD [23]. Also, the AIP index could help determine the risk of T2DM in middle-age people [42]. Moreover, VAI plays a significant role in estimating the visceral fat content and identifying high-risk people for cardio-metabolic diseases, including MUO and MUNO phenotype [43, 44]. Additionally, the LAP index can detect the risk of CVD and metabolic syndrome [45, 46]. Also, the TyG index, which is a product of plasma glucose and TG, has a positive association with cardiovascular risk factors, such as increased SBP and DBP, FPG, and TG, and a negative association with HDL-c [47]. Besides, in patients with Hypertension, CMI is associated with new-onset CVD; this index helps identify high-risk people for CVD [48]. Overall, all cardio-metabolic indices can assist in the early identification of high-risk people, especially for CVD. It can be concluded that all metabolic obesity phenotypes that are related to these indices are associated with cardio-metabolic diseases. In a study conducted in Ahvaz, Iran, it was found that AIP, LAP, CMI, and consequently, the risk of CVD were significantly higher in the MUO and MUNW phenotypes compared to metabolically healthy phenotypes. They proposed a more significant correlation of metabolic health with CVD compared to the correlation of obesity with CVD [39]. The stable MUO phenotype basically increases the risk of all vascular disease basically; this risk is intensified in transition from the MHO phenotype to the MUO phenotype. In other studies, the risk of CVD was also higher in metabolically unhealthy individuals compared to metabolically healthy phenotypes. However, this finding is contrary to previous research, which claimed that metabolic status had no value in identifying CVD risk compared to BMI [49–51]. In our study, history of IHD, MI, and Stroke was significantly higher in metabolically unhealthy phenotype as compared to metabolically healthy individuals. In a previous study, obesity without an unhealthy metabolic status did not increase the risk of CVD during a 15.9-year follow-up [52]. As mentioned earlier, the MHO phenotype is associated with a normal subcutaneous adipose tissue, decrease visceral fat content, and reduced fat deposition in the liver, leading to a lower risk of metabolic abnormalities and heart diseases compared to unhealthy metabolic phenotypes [7, 53]. Nevertheless, in the most extensive Chinese prospective cohort study, an association was observed between the MHO phenotype and the increased risk of major vascular events [49]. Persistent MHO was associated with a higher risk CVD during a 10-year follow-up compared to the MHNW phenotype [54]. Evidence shows that longer exposure to metabolically unhealthy conditions increases the risk of CVD [49]. Considering the increased risk of cardio-metabolic disease in the MUNO phenotype, this phenotype should not be interpreted as risk–free due to the absence of obesity. Generally, maintenance of metabolic health is the main goal of cardio-metabolic diseases prevention programs, even for individuals with normal weights. Therefore, people who are metabolically healthy should maintain their metabolic health, blood glucose, blood pressure, and lipid profile at normal levels. Lifestyle interventions should be also encouraged for high-risk populations to prevent cardio-metabolic diseases [49].

### Strengths and limitations

This study had several strengths. First, it had a large sample size, as we analyzed the Hoveyzeh Cohort Study data. Second, measurements of liver indices for steatosis and NAFLD could partially eliminate the need for ultrasound. This study also had some limitations. First, the cross-sectional design of the study restrained us from defining causal inference. The Neyman’s bias or incidence-prevalence bias is also plausible; this type of bias occurs depending on the time of including cases in a research study and is more likely to affect long-term diseases than short-term conditions. Fatal and short episodes, mild/silent episode, and cases with disappearing evidence of exposure following disease onset were not included. Therefore, the selected sample did not include the mentioned cases. Careful selection of samples is crucial for developing an accurate understanding of a diseases and its causes. Also, using incident cases rather than prevalent cases can avoid Neyman’s bias. Second, although this study was performed on multi-ethnic adults, the number of adults from different ethnic group was small, and they only lived in the southwest of Iran; therefore, the present results cannot be extended to all ages and ethnicities. Further studies are suggested on different ethnic populations and regions. Third, the definition of metabolic health varies in different studies, and for this reason, it is not simple to make accurate comparisons. Finally, the family relations of the participants can be considered as a confounding factor.

## Conclusions

Based on the present results, individuals with the MUNO phenotype were exposed to a higher risk of CVD compared to those with the MHO phenotype; therefore, a healthy lifestyle and follow-up are essential for these individuals. The association of a metabolically unhealthy status with CVD was more prominent than the association of obesity with CVD. The highest grades of steatosis and NAFLD were found in the MUO phenotype. After adjustment for age, sex, physical activity, and education years, VAI had the highest OR for MUNO and MUO compared to the MHNO phenotypes. VAI can be introduced as the best index for CVD risk assessment. The ANI was also associated with a reduced risk of MUO, MUNO, and MHO phenotypes.

## Electronic supplementary material

Below is the link to the electronic supplementary material.

Supplementary Material 1

## Data Availability

Data used in this study can be obtained from the corresponding author upon reasonable request.

## Abbreviations

MHO: Metabolically Healthy Obesity
MHNO: Metabolically Healthy Non-Obese
MUO: Metabolically Unhealthy Obesity
MUNO: Metabolically Unhealthy Non-Obese
NCEP ATP III criteria: National Cholesterol Education Program and Adult Treatment Panel III criteria
WHR: Waist-to-Hip Ratio
BAI: Body Adiposity Index
VAI: Visceral Adiposity Index
AIP: Atherogenic Index of Plasma
LAP: Lipid Accumulation Product
CMI: Cardio-Metabolic Index
LCI: Lipoprotein Combined Index
TIMI risk Index: Thrombolysis risk In Myocardial Infarction Index
HSI: Hepatic Steatosis Index
ANI (ALD/NAFLD): Alcoholic Liver Diseases/Non-Alcoholic Fatty Liver Diseases Index
