# Supplementary material for: The Association between Cardio-metabolic and hepatic indices and anthropometric measures with metabolically obesity phenotypes: a cross-sectional study from the Hoveyzeh Cohort Study
Source: BMC Endocr Disord. 2023 May 29;23:122. doi: 10.1186/s12902-023-01372-9 (PMC10226206; doi:10.1186/s12902-023-01372-9)
Supplement: Supplementary file 1 — Supplementary Material 1 [file 12902_2023_1372_MOESM1_ESM.doc]

**Supplementary file.** Spearman Correlation Coefficient between Cardio-metabolic and Hepatic indices and Anthropometric measures in a cross sectional study of Hoveyzeh Cohort

| **Variables** | **WHR** | **WHtR** | **BMI** | **BAI** | **VAI** | **WWI** | **AIP** | **LAP** | **CMI** | **LCI** | **TyG Index** | **TyG-BMI** | **TyG-WC** | **TIMI** | **ANI** | **HSI** |
| --- | --- | --- | --- | --- | --- | --- | --- | --- | --- | --- | --- | --- | --- | --- | --- | --- |
| **WHR** | 1 |  |  |  |  |  |  |  |  |  |  |  |  |  |  |  |
| **WHtR** | 0.539^b^ | 1 |  |  |  |  |  |  |  |  |  |  |  |  |  |  |
| **BMI** | 0.308 ^b^ | 0.841 ^b^ | 1 |  |  |  |  |  |  |  |  |  |  |  |  |  |
| **BAI** | 0.031 | 0.823 ^b^ | 0.730 ^b^ | 1 |  |  |  |  |  |  |  |  |  |  |  |  |
| **VAI** | 0.327^b^ | 0.234 ^b^ | 0.208 ^b^ | 0.045 ^b^ | 1 |  |  |  |  |  |  |  |  |  |  |  |
| **WWI** | 0.596 ^b^ | 0.756 ^b^ | 0.309 ^b^ | 0.592 ^b^ | 0.172 ^b^ | 1 |  |  |  |  |  |  |  |  |  |  |
| **AIP** | 0.273 ^b^ | 0.102 ^b^ | 0.161 ^b^ | -0.097 ^b^ | 0.970 ^b^ | -0.011 | 1 |  |  |  |  |  |  |  |  |  |
| **LAP** | 0.500 ^b^ | 0.596 ^b^ | 0.567 ^b^ | 0.345 ^b^ | 0.843 ^b^ | 0.385^b^ | 0.775 ^b^ | 1 |  |  |  |  |  |  |  |  |
| **CMI** | 0.371 ^b^ | 0.296 ^b^ | 0.324 ^b^ | 0.073 ^b^ | 0.978 ^b^ | 0.141^b^ | 0.976 ^b^ | 0.874 ^b^ | 1 |  |  |  |  |  |  |  |
| **LCI** | 0.260 ^b^ | 0.129 ^b^ | 0.147 ^b^ | -0.035^b^ | 0.756 ^b^ | 0.055^b^ | 0.769 ^b^ | 0.692 ^b^ | 0.762 ^b^ | 1 |  |  |  |  |  |  |
| **TyG Index** | 0.356 ^b^ | 0.190 ^b^ | 0.187 ^b^ | -0.029^b^ | 0.833 ^b^ | 0.113^b^ | 0.834 ^b^ | 0.792 ^b^ | 0.838 ^b^ | 0.838 ^b^ | 1 |  |  |  |  |  |
| **TyG-BMI** | 0.393 ^b^ | 0.797 ^b^ | 0.931 ^b^ | 0.622 ^b^ | 0.465 ^b^ | 0.309^b^ | 0.424 ^b^ | 0.776 ^b^ | 0.571 ^b^ | 0.376 ^b^ | 0.504 ^b^ | 1 |  |  |  |  |
| **TyG-WC** | 0.636 ^b^ | 0.773 ^b^ | 0.743 ^b^ | 0.435 ^b^ | 0.607 ^b^ | 0.480^b^ | 0.542 ^b^ | 0.883 ^b^ | 0.681 ^b^ | 0.482 ^b^ | 0.657 ^b^ | 0.891 ^b^ | 1 |  |  |  |
| **TIMI** | 0.340 ^b^ | 0.140 ^b^ | -0.097 ^b^ | -0.010 ^b^ | 0.035 | 0.369^b^ | -0.005 | 0.055 | 0.021 | 0.048 ^a^ | 0.106 ^a^ | -0.045 ^b^ | 0.120 ^b^ | 1 |  |  |
| **ANI** | -0.148 ^b^ | -0.626 ^b^ | -0.527 ^b^ | -0.673 ^b^ | -0.124^b^ | -0.488 ^b^ | 0.013 | -0.347^b^ | -0.115^b^ | -0.002 | -0.084 | -0.490 | -0.408 ^b^ | 0.024 | 1 |  |
| **HIS** | 0.191^b^ | 0.691^b^ | 0.564 ^b^ | 0.732 ^b^ | 0.176^b^ | 0.560^b^ | 0.021 | 0.411^b^ | 0.160 ^b^ | 0.047^b^ | 0.259 ^b^ | 0.591^b^ | 0.530^b^ | 0.044 ^b^ | -0.685^b^ | 1 |

^a^ correlation is significant at the 0.05 level

^b^ correlation is significant at the 0.01 level

WHR: Waist-To-Hip Ratio; WHtR: Waist-To-Height Ratio; BMI: Body Mass Index; BAI: Body Adiposity Index; VAI; Visceral Adiposity Index; WWI: Weight-adjusted Waist Index; AIP: Atherogenic Index of Plasma; LAP: Lipid Accumulation Product; CMI: Cardio-metabolic index; LCI: Lipoprotein Combine Index; TIMI: TIMI risk index; ANI: ALD/NAFLD Index; HSI: Hepatic Steatosis Index
